# Colorectal cancer screening with faecal immunochemical test: Patterns of participation

**DOI:** 10.1177/09691413231188275

**Published:** 2023-07-18

**Authors:** Hanna Heyman, Johannes Blom, Deborah Saraste

**Affiliations:** Department of Surgery, Södersjukhuset, Stockholm and Department of Clinical Science and Education, 27106 Karolinska Institutet, Stockholm, Sweden

**Keywords:** colorectal cancer screening, faecal immunochemical test, FIT, participation patterns, uptake

## Abstract

**Objective:**

To evaluate participation and participation patterns in a population-based screening programme for colorectal cancer (CRC) using the faecal immunochemical test (FIT).

**Methods:**

All individuals invited to three consecutive screening rounds in the population-based CRC screening between October 2015 and December 2020 in the Stockholm–Gotland Region, Sweden were included. Patterns of participation were assessed.

**Results:**

The study included 26 541 individuals which resulted in 79 623 screening events. The overall uptake rate was 71.5% and women had a significantly higher participation rate. The participation rate increased significantly between the first and third screening round for both men and women, and the increase was larger among men than women (66.1 to 70.7% *vs*. 73.1 to 75.4%). In total, 80.9% participated at least once. Consistent participation was the most common participation pattern (61.0%). The probability of attending all three consecutive rounds after initial participation was 87.7%. Over the three rounds, 17.4% participated after a reminder letter. Screening individuals attending after a reminder letter had a higher proportion of drop-outs in the following screening round compared to initial participants (15.4% vs 6.2%).

**Conclusion:**

A constant and high participation rate was observed in population-based FIT-screening for CRC. Initial participation was a strong predictor for continuous participation. The need for a reminder letter before participation was a risk factor for subsequent drop-out.

## Introduction

The aim of population-based cancer-screening is to reduce disease-specific mortality by early detection and treatment.^1^ Several population-based randomised clinical trials have demonstrated the efficacy of colorectal cancer (CRC) screening using faecal occult blood test (FOBT), with a relative disease-specific mortality reduction of 16% with FOBT.^2^

There are differences in participation among those invited to screening for CRC. Women and older age groups participate to a higher degree than men and younger invitees, and participation decreases with increased social deprivation.^3,4^ During the last decade, most screening programmes have adopted the faecal immunochemical test (FIT), which is a quantitative test using antibodies specific to the globin part of human haemoglobin. FIT is recommended by the EU guidelines and earlier studies have shown a significant increase in participation rates with FIT compared to guaiac-based FOBT (gFOBT).^5–8^

In the population-based screening programme for CRC in the Swedish region of Stockholm-Gotland, the test-kit with gFOBT (Hemocult^®^) was replaced by FIT (OC-Sensor^®^, Eiken, Japan) in 2015 and the participation rate increased from approximately 56% to 68%.^9^

Earlier studies evaluating participation patterns with gFOBT demonstrated that initial participation was a strong predictor for participation in the next screening round, and that consistent participation was the most common pattern of participation, although with lower participation rates overall (60%).^10,11^ To identify groups within the screening programme who might need more specific interventions to increase their participation, it is necessary to get a better understanding of participation patterns.

The aim of this study was to describe the patterns of participation for repeated screening rounds with FIT in the Swedish region of Stockholm–Gotland and to identify screening behaviour that could predict non-participation in subsequent screening rounds.

## Methods

The population-based CRC screening programme of the Stockholm-Gotland region started in 2008. The programme was fully rolled out in 2013 and invites approximately 115 000 new individuals aged 60–69 years each year. The age interval of screened individuals was expanded to 70 years in 2021 and it will be implemented in all Swedish regions by 2026.^3^ The invitees receive a test-kit to detect faecal haemoglobin every second year. On the 1^st^ of October 2015 the test-kit changed from gFOBT (Hemocult) to FIT (OC-Sensor), which was introduced to all invited individuals in the region of Stockholm/Gotland on the same date. The cut-off for positive FIT was set to 80 µg/g for men and 40 µg/g for women. The organisation of the screening programme is centralised to the Regional Cancer Centre and does not involve primary care physicians. Test-kits are sent to the screening invitees with a free return envelope and a reminder letter is sent if the test-kit is not returned within eight weeks. A returned test-kit is defined as participation. The analyses are centralised to one laboratory, until 1 January 2018 at Medilab, Täby, Stockholm and thereafter at Karolinska University, Solna, Stockholm. Participants with a positive test result are invited to a follow-up diagnostic colonoscopy. The colonoscopy is only registered if it is completed. If no cancer or polyps are diagnosed, the participant will receive a new screening invitation after 2 years.

Individuals invited for three consecutive rounds of CRC screening with FIT between 1 October 2015 and 31 December 2020 were included in the study. Participants who were diagnosed with adenomatous polyps or CRC in their first or second screening round were excluded from the study, as they are excluded from the screening programme and followed up in other programmes.

Data were extracted from the Register for Colorectal Cancer Screening Stockholm and the Swedish Register for Colonoscopy and Colorectal Cancer screening (SweReKKS). The registers are centrally administered and collect all data from the screening process, such as FIT values, gender, age, colonoscopy findings and complications.

Patterns of participation were categorised into eight different patterns for the three screening rounds; Y (Yes) equals a returned test-kit and N (No) a non-returned test-kit. Drop-out was defined as an initial participation with FIT followed by non-participation in subsequent screening rounds.

The participation rates were calculated as the number of participants divided by all sent invitations. The proportion of participation after reminder was defined as the number of participants after reminder divided by all participants. The differences in participation between men and women were calculated using a Chi^2^ test of proportions at a statistical significance level of 5% (*p* = 0.05).

The study was approved by the Regional Ethical Review Board, Stockholm (2016/1927-31/4, 2018/1311-32 and 2020-04417).

## Results

**Figure 1.**
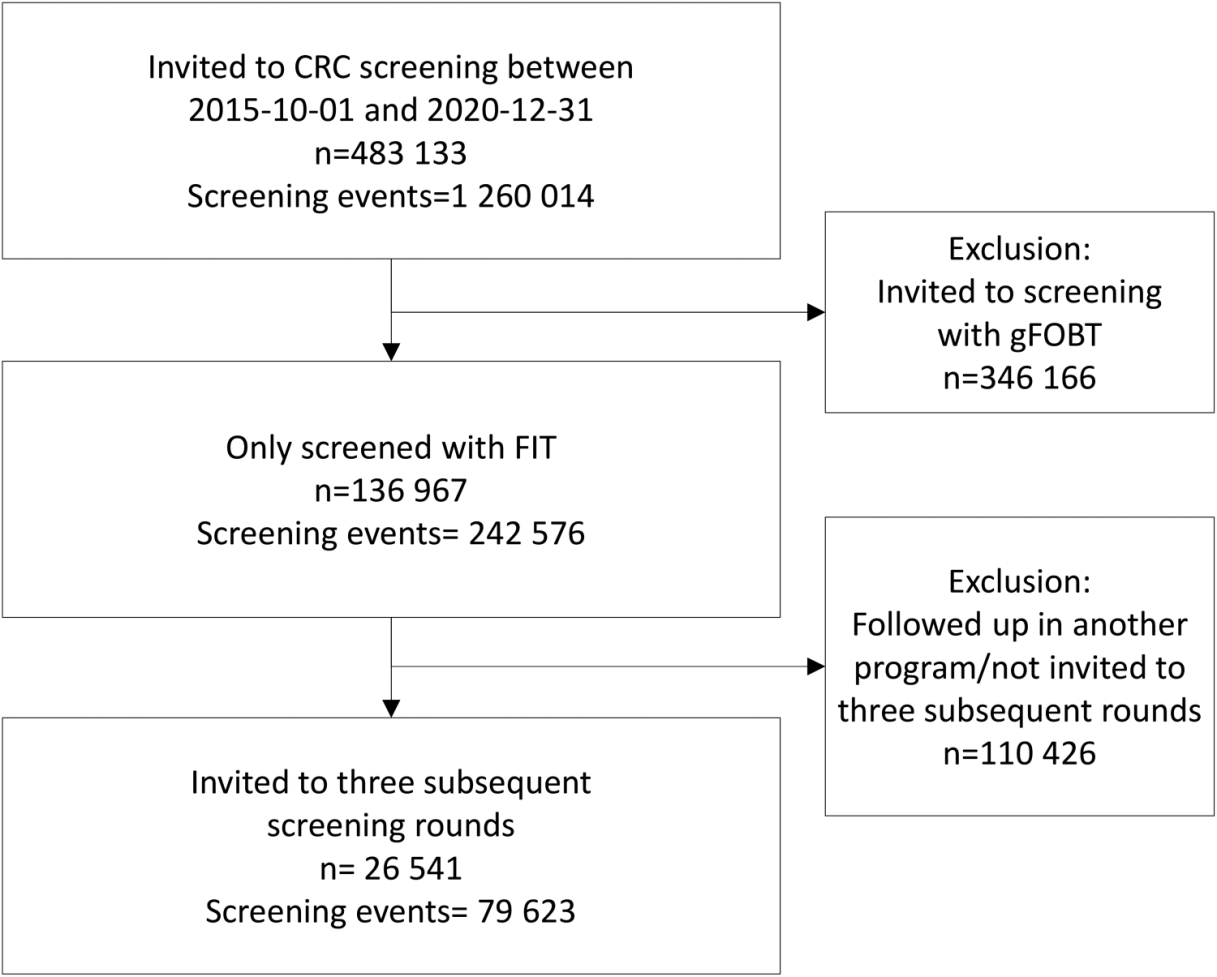
Study population.

**Figure 2.**
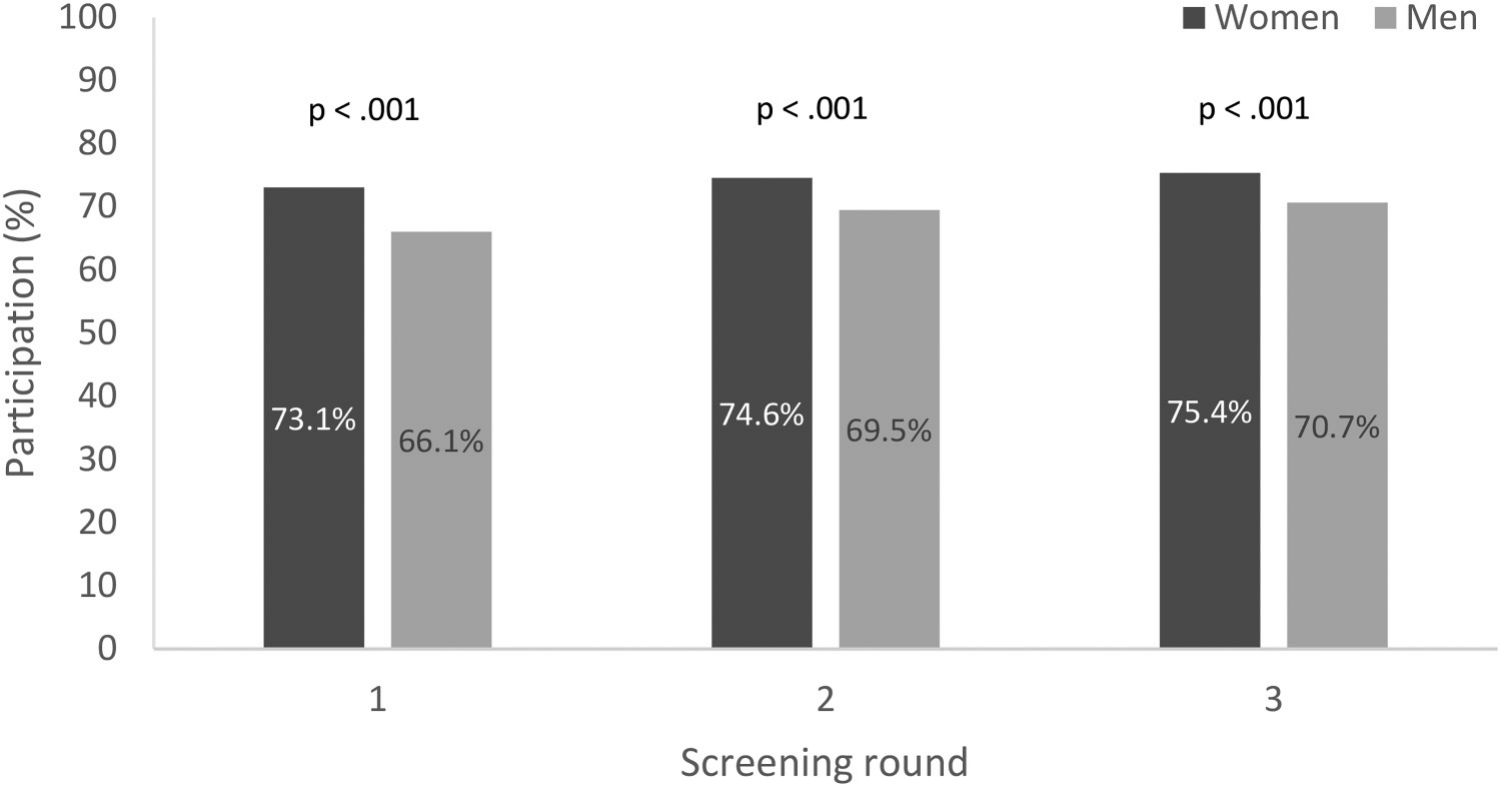
Participation rates per screening round by gender.

During the study period, 26 541 individuals were included (Figure 1). The population was born between 1951 and 1956 and 86.6% were born in 1956. The overall participation rate was 71.5% during the follow-up period (Table 1). Women had a statistically significantly higher participation rate in each round (Figure 2). Both men (*p* < 0.001) and women (*p* < 0.001) had a significant increase in participation rate between the first and third screening round. The study excluded all individuals who had a polyp or cancer in the first or second screening round, and therefore the positivity rate was higher in the third round compared to the earlier rounds in this cohort. The positivity rate for all invitees born in 1956 for each separate round was 2.4% in the first screening round, 2.0% in the second, and 2.4% in the third.

**Table 1.** Participation over three subsequent CRC screening rounds.

Screening round		Invited, *n*	Participated, *n* (%)	Participated after reminder, *n* (%)	Positive FIT, *n* (%)	Colonoscopy after positive FIT, *n* (%)
1	Women	13279	9706 (73.1)	1504 (15.5)	175 (1.8)	150 (85.7)
1	Men	13262	8761 (66.1)	1624 (18.5)	122 (1.4)	105 (86.1)
1	Total	26541	18467 (69.6)	3128 (16.9)	297 (1.6)	255(85.9)
2	Women	13279	9906 (74.6)	1759 (17.8)	154 (1.6)	135 (87.7)
2	Men	13262	9213 (69.5)	1668 (18.1)	132 (1.4)	97 (73.5)
2	Total	26541	19119 (72.0)	3427 (17.9)	286 (1.5)	232 (81.1)
3	Women	13279	10007 (75.4)	1685 (16.8)	243 (2.4)	198 (81.5)
3	Men	13262	9373 (70.7)	1684 (18.0)	225 (2.4)	175 (77.8)
3	Total	26541	19380 (73.0)	3369 (17.4)	468 (2.4)	373 (79.7)
All rounds	Women	39837	29619 (74.4)	4948 (16.7)	572 (1.9)	483 (84.4)
All rounds	Men	39786	27347 (68.7)	4976 (18.2)	479 (1.8)	377 (78.7)
All rounds	Total	79623	56966 (71.5)	9924 (17.4)	1051 (1.8)	860 (81.8)

Of all those participants with a positive FIT, 81.8% completed a subsequent colonoscopy. The colonoscopy uptake decreased during the study period (Table 1). The last screening round took place between January 2019 and December 2020, which coincided with the COVID-19 pandemic. The colonoscopy uptake in our study was compared to the other invitees invited to the screening programme during these years. The colonoscopy uptake in 2020 was 86.9% for the individuals invited to their first screening round, 84.2% for the second, and 79.8% for the third screening round. For the third round in 2021, the uptake was 84.7%.

**Figure 3.**
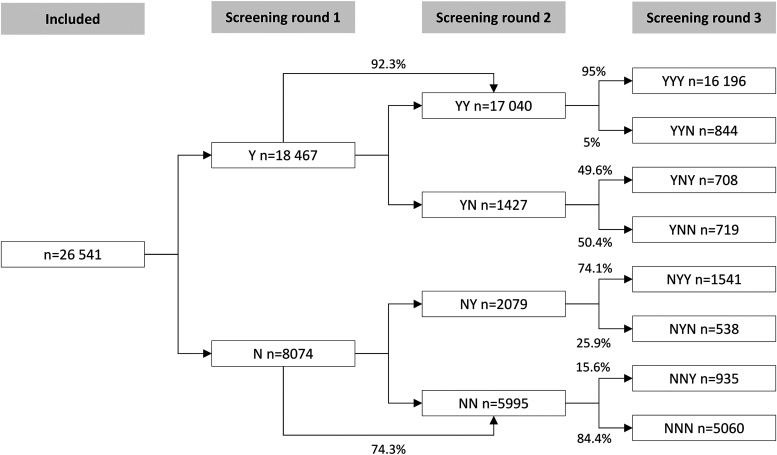
Participation patterns and probability to participate in following screening rounds.

The most common pattern of participation (61.0%) was consistent participation (Table 2 and Figure 3). In total, 80.9% participated at least once. Among women, a greater proportion were consistent participants compared to men: 64.6% vs 57.4% (*p* < 0.001). After an initial non-participation, 11.4% became participants in screening round two or three (NYY, NYN, NNY) (12.8% and 9.9% of men and women, respectively).

**Table 2.** Patterns of participation in three complete screening rounds.

Participation pattern	Total, *n* (%)	Women, *n* (%)	Men, *n* (%)
YYY*	16196 (61.0)	8579 (64,6)	7617 (57,4)
YYN	844 (3,2)	420 (3,2)	424 (3,2)
YNY	708 (2,7)	353 (2,7)	355 (2,7)
YNN	719 (2,7)	354 (2,7)	365 (2,8)
NYY	1541 (5,8)	662 (5,0)	879 (6,6)
NYN	538 (2,0)	245 (1,9)	293 (2,2)
NNY	935 (3,5)	413 (3,1)	522 (3,9)
NNN**	5060 (19,1)	2253 (17,0)	2807 (21,2)
Total	26541	13279	13262

*Y (Yes) = participation

**N (No) = non-participation

The probability of attending all three consecutive screening rounds if attending the first was 87.7% (Figure 3). The probability of attending the last screening round if not participating in the first was 30.7%. After an initial non-participation, the probability of continuous non-participation in the following rounds was 62.7%.

A reminder letter was sent to invitees who had not returned the test-kits within 8 weeks. In total, 17.4% of all participation occurred after a reminder letter. The proportion of drop-outs in the second screening round was higher among those needing a reminder letter in the first round compared to those who participated without a reminder: 15.4% (482/3128) vs 6.2% (945/15339). Similar numbers were seen between the second and third screening round: 15.6% (535/3427) vs 5.4% (847/15692), showing a higher drop-out proportion among those who participated after the reminder of the previous round. Of all reminders sent out, 30% resulted in participation.

## Discussion

The overall participation rate over three rounds was 71.5% and increased significantly from the first to the third round. Women had a significantly higher participation rate compared to men, but the difference decreased with the number of rounds.

The most common participation pattern with FIT was consistent participation. However, the proportion of consistent participants was greater in this study (61.0%) compared to a previous study evaluating screening with gFOBT where the continuous participation rate was 49.8%.^11^ The increase in uptake following the introduction of FIT is hence due to less consistent non-participants and a higher proportion of consistent participants. The higher participation rates with FIT have been explained partly by its more user-friendly characteristics, for example needing only one instead of three stool samples per screening round.^12^

Participation in the first screening round was a strong predictor for continuous uptake in screening with FIT (87.7%) which confirms the results from previous studies on gFOBT.^11,13^ Therefore, to increase uptake on a long-term basis it is crucial to achieve a high participation rate at the first screening invitation.

The overall participation rate was 71.5% and is higher in comparison with most other gFOBT/FIT-based CRC screening programmes with reported uptakes of 45% to 65%.^14–16^ This study showed that the difference in participation rates between men and women continues to be statistically significant throughout three consecutive screening rounds. Consistent with previous findings from the English and Australian screening programmes, our study highlights that men were more likely to become participants in later screening rounds, as compared to women.^13,17^ The larger proportion of late entrants among men shows that repeated invitation in the screening programme reduces gender differences in uptake. One possible explanation for the differences could be that Swedish women have experienced breast and cervical screening before invitation to CRC screening. In contrast, men have not yet been exposed to screening when they receive their first invitation. At the age of 65, Swedish men are invited to an abdominal aortic aneurysm screening, with a participation rate of 78%, which might have a positive influence on attitude to screening among Swedish older men.^18^

As we wanted to study participation over three consecutive rounds with FIT, the time needed limited the cohort to a narrow age group. The positivity rate increased over consecutive rounds in this study, which can be explained by the inclusion criteria since all individuals with cancer or polyps in the first two rounds were excluded. Therefore, it cannot be interpreted as a tendency of increasing positivity rate over time.

In our study, 17.4% participated after receiving a reminder letter, and this effect was greatest among men in the first round (18.5%). Since participation in the first screening round is a strong predictor for continuous participation this indicates that a reminder is effective to improve participation among men who have a lower overall participation rate compared to women. Similar to our findings, the Dutch FIT screening programme had a high participation rate after reminder letter (12%), but the benefit of a reminder letter was only seen in initial participation and did not remain effective over multiple rounds, as demonstrated in our study.^16^

Within the group that participated after a reminder letter, there was a higher proportion of drop-outs in the next round. This finding defines a subgroup who have a history of being receptive to reminders but who subsequently drop out. To improve screening uptake, this group may benefit from a specific intervention in their second round, such as an extra reminder, to prevent future non-participation.

The overall colonoscopy uptake after a positive FIT was 81.8% which is lower than the uptake of 85% described in the Dutch and Taiwanese programmes, but higher than the participation described in an Italian programme.^19–21^ Despite the increasing FIT participation by screening round, the colonoscopy uptake was reduced over multiple screening rounds. Scottish data, evaluating participation with gFOBT, demonstrated an increase in colonoscopy uptake over multiple screening rounds, despite a participation in the faecal test of around 50%.^22^

The reason for the decline in colonoscopy uptake is not clear, but the third screening round partly coincided with the COVID-19 pandemic. The temporary reduction in colonoscopy uptake might have been a consequence of the pandemic even though it did not influence participants invited to other screening rounds the same year. This should be reevaluated in coming screening rounds that are not affected by pandemic restrictions. A possible explanation for the decreasing colonoscopy uptake over multiple screening rounds could be that invitees decline a second colonoscopy if the previous one was negative.

In conclusion, our study demonstrated a constant and high participation rate of 71.5% in population-based screening for CRC, and a colonoscopy uptake of 81.8% after a positive FIT. Initial participation was a predictor of continuous participation, and the need for a reminder letter before participation was a risk factor for subsequent drop-out. Further efforts should be made to increase initial participation and to intervene to prevent drop-out from the programme.
